# One Spot—Two Sensors: Porous Silicon Interferometers in Combination With Gold Nanostructures Showing Localized Surface Plasmon Resonance

**DOI:** 10.3389/fchem.2019.00593

**Published:** 2019-09-04

**Authors:** Ruth Fabiola Balderas-Valadez, Robin Schürmann, Claudia Pacholski

**Affiliations:** Institute of Chemistry, University of Potsdam, Potsdam, Germany

**Keywords:** porous silicon, interferometry, gold nanostructures, surface plasmon resonance, optical sensor

## Abstract

Sensors composed of a porous silicon monolayer covered with a film of nanostructured gold layer, which provide two optical signal transduction methods, are fabricated and thoroughly characterized concerning their sensing performance. For this purpose, silicon substrates were electrochemically etched in order to obtain porous silicon monolayers, which were subsequently immersed in gold salt solution facilitating the formation of a porous gold nanoparticle layer on top of the porous silicon. The deposition process was monitored by reflectance spectroscopy, and the appearance of a dip in the interference pattern of the porous silicon layer was observed. This dip can be assigned to the absorption of light by the deposited gold nanostructures leading to localized surface plasmon resonance. The bulk sensitivity of these sensors was determined by recording reflectance spectra in media having different refractive indices and compared to sensors exclusively based on porous silicon or gold nanostructures. A thorough analysis of resulting shifts of the different optical signals in the reflectance spectra on the wavelength scale indicated that the optical response of the porous silicon sensor is not influenced by the presence of a gold nanostructure on top. Moreover, the adsorption of thiol-terminated polystyrene to the sensor surface was solely detected by changes in the position of the dip in the reflectance spectrum, which is assigned to localized surface plasmon resonance in the gold nanostructures. The interference pattern resulting from the porous silicon layer is not shifted to longer wavelengths by the adsorption indicating the independence of the optical response of the two nanostructures, namely porous silicon and nanostructured gold layer, to refractive index changes and pointing to the successful realization of two sensors in one spot.

## Introduction

Optical biosensors are composed of a biological layer and a light-interacting transducer. Here, the biological layer is responsible for guaranteeing the selective determination of target molecules and the transducer for converting the recognition event into a measurable signal. Optical signal transduction is most often achieved either by interferometry, which can be exploited in porous silicon sensors for example (Pacholski, 2013), or by localized surface plasmon resonance (LSPR) occurring in gold nanoparticle based sensors (Willets and Van Duyne, 2007). Both sensor types detect changes in the refractive index and provide different advantages and disadvantages concerning their performance, which have been critically reviewed (Kedem et al., 2012).

Interference based optical sensing relies on superimposition of light waves leading to constructive and destructive interference in order to detect analytes. In the case of porous silicon sensors, light rays are reflected at the interfaces of the porous silicon layer resulting in the appearance of an interference pattern, so-called Fabry-Pérot fringes, in reflectance spectra (Janshoff et al., 1998). The Fabry-Pérot fringes shift their position on the wavelength scale as a function of the effective refractive index of the porous silicon layer (= porous silicon + material filling the pores). This simple optical signal transduction, the high surface area, and the convenient surface chemistry of porous silicon are most favorable prerequisites for developing highly sensitive optical sensors. Furthermore, key properties of the porous silicon, such as layer thickness and porosity, can be satisfyingly controlled by the most often utilized fabrication method—electrochemical etching (Zhang, 2005). Tailor-made fabrication strategies for more sophisticated devices (Rodriguez et al., 2019), well-thought through surface functionalization methods (Mariani et al., 2018b), and smartly chosen conditions for detecting target analytes (Arshavsky-Graham et al., 2017; Mariani et al., 2018a) provided optical porous silicon sensors whose performance can compete with other optical sensing platforms. A comprehensive review has recently been published (Arshavsky-Graham et al., 2019).

Another route to porous silicon is metal-assisted etching, in which metal nanostructures catalyze the dissolution of silicon in solutions containing hydrofluoric acid and an oxidant (Huang et al., 2011). Here, metal nanostructures are often deposited on silicon by galavanic displacement reactions, i.e., by immersion of silicon wafers in hydrofluoric acid solutions containing metal ions. Deposition of metal nanostructures on porous silicon can be obtained in the same way, and the resulting materials have intensively been investigated for surface-enhanced Raman spectroscopy, a technique exploiting localized surface plasmon resonance (LSPR).

LSPR is a charge-density oscillation that may exist at the interface of two media with dielectric constants of opposite sign, e.g., metallic nanoparticles over dielectric, provoking a selective absorption and scattering of photons (Hicks et al., 2005; Stewart et al., 2008). Both absorption and scattering strongly depend on the size, shape, and composition of the nanostructure, as well as the refractive index of the surrounding medium (Guo et al., 2015). Consequently, the optical properties can be tailor-made by choosing the appropriate design of the metal nanostructure for the desired application, which include energy harvesting (Atwater and Polman, 2010), medicine (Zhang H. et al., 2018), surface enhanced spectroscopy and optical sensors (Mayer and Hafner, 2011). Optical sensors utilizing LSPR for signal transduction detect changes in the refractive index (*n*) in close proximity to the sensor surface (up to ~10 nm), i.e., changes in *n* induced by absorption or binding of chemical or biological species on the metallic nanostructure. The success of LSPR sensors can be traced back to their simple optical read-out method, well-established functionalization protocols, and versatile fabrication techniques ranging from bottom-up to top-down strategies.

Moreover, as already indicated before, another important characteristic of plasmonic nanostructures showing LSPR is their ability to enhance spectroscopic signals such as Raman scattering or IR absorption (Jahn et al., 2016). Raman scattering is a very useful tool in the field of analytical chemistry and biology. However, the commonly weak Raman signals and the consequently relative high amount of analyte needed for Raman detection limited its application. The attachment to or deposition of the analyte on metal nanostructures can improve the Raman signal and reduce the quantity of analyte required. This technique is called surface enhanced Raman spectroscopy (SERS). The reproducibility and enhancement of SERS signals depend strongly on the position, alignment, size and morphology of the metallic nanoparticles (Fan et al., 2011). Highly homogeneous and reproducible substrates for SERS have been prepared using e.g., electron-beam (Yue et al., 2012) or focused ion beam lithography (Gao et al., 2012). These techniques require sophisticated laboratory equipment. More cost-efficient approaches based on colloidal lithography (Balderas-Valadez et al., 2018; Zhang T. et al., 2018) or biological templates (Wu et al., 2018) have been investigated for fabricating SERS substrates, but their wide spread use is challenged by the requested reproducibility of SERS enhancement—over the whole substrate surface and in different batches of SERS substrates. However, SERS is a powerful technique, which was already employed for detecting a variety of analytes ranging from explosives (Ben-Jaber et al., 2017) to bacteria (Wang et al., 2015).

Porous silicon covered with metal nanostructures was mainly investigated for SERS applications (Bandarenka, 2016) until now. Here, porous silicon served as highly attractive material for preparing as well as supporting metal nanostructures and often did not significantly contribute to the optical signal transduction, which is based on localized surface plasmon resonance (Virga et al., 2012). Initial attempts have been made to take advantage of sensors consisting of both porous silicon and gold nanoparticles. For example, Jiao et al. (2010) developed a dual-mode sensor in which gold nanoparticles were covalently bonded to porous silicon using silane chemistry. This sensor was able to detect benzene-thiol binding to the gold nanoparticles both interferometrically and by means of SERS. In addition, optical sensors based on porous silicon microcavities decorated with gold nanoparticles have been used to detect DNA by surface enhanced fluorescence (Wang and Jia, 2018).

Only recently, a new generation of optical sensors combining LSPR and interferometry receives increasing interest (Schmidt et al., 2012; Ameling et al., 2014). The attractiveness of this strategy is driven by both the progress in basic research on the optical properties of metamaterials and the realization of multimode optical sensors. Different optical phenomena have been observed for materials combining interferometric/photonic materials with plasmonic nanostructures, e.g., splitting of Fabry-Pérot fringes (Liu et al., 2015). Furthermore, an improved performance of optical sensors based on merged photonic/plasmonic structures in comparison to sensors consisting of only one component have been proposed. These sensors can be composed of 1D porous silicon photonic crystal structures covered with a thin metal layer, which show so-called Tamm resonances in their reflection spectra (Juneau-Fecteau and Fréchette, 2018; Ahmed and Mehaney, 2019). The full potential of this type of optical sensors has not yet been exploited.

In this work, an optical sensor facilitating both interferometric and LSPR signal transduction at the same time is presented. The sensor was fabricated by electrochemically etching of silicon and subsequent gold nanoparticle deposition. The reflectance spectrum of this sensor showed Fabry-Pérot fringes with a broad dip in the interference pattern, which could be assigned to LSPR. The optical responses of the sensor to refractive index changes were thoroughly investigated and support the hypothesis that interferometric and LSPR sensing can be performed independently using the same spot on the sensor. Furthermore, the gold nanostructure on top of the porous silicon layer has successfully been exploited for SERS spectroscopy providing additional information on the target analyte.

## Materials and Methods

### Materials

Silicon wafers (*p*-type, 0.001–0.002 Ω cm, <100>) were purchased from Siegert Wafer GmbH (Germany). Hydrofluoric acid (48%) was supplied by Merck and ethanol (99.8%) by Carl Roth GmbH + Co. KG (Germany). The growth of gold nanostructures was carried out with ethanol (96 %) obtained from VWR International GmbH (Germany). HAuCl_4_ · 3 H_2_O (99.99%) was supplied by Alfa Aesar (Thermo Fisher (Kandel) GmbH, Germany). Toluene was also purchased from VWR International GmbH (Germany) and thiol terminated polystyrene (3,1400 g/mol) was obtained from Polymer Source, Inc (Canada).

### Methods

#### Fabrication of Porous Silicon Films

Porous silicon films were fabricated by electrochemically etching. For this purpose, silicon wafers (single side polished, p-type, resistivity: 0.001–0.002 Ω cm, thickness: 552 ± 25 μm) were cut into smaller pieces of roughly 1.6 × 1.6 cm using a diamond cutter. A small piece of silicon was then fixed in a custom-made Teflon etching cell (Sailor, 2012). Here, an appropriately prepared aluminum foil served as back contact for the silicon and a platinum ring electrode was used as counter electrode. The etching cell was filled with an etching solution composed of a 1:1 (v:v) mixture of hydrofluoric acid (49%) and ethanol. The porous silicon films were obtained by applying a current density of 132.63 mA cm^−1^ for 67 s using a Kepco ATE25-2M power supply (USA). Afterwards, the etching solution was removed with a plastic pipette and the porous silicon film was washed with ethanol several times. Finally, the porous silicon film was blown dry with a stream of nitrogen.

#### Deposition of Gold Nanostructure on Porous Silicon Films

Freshly etched porous silicon films were directly immersed at room temperature (25–30°C) in 3 mL of an aqueous solution containing 2 mM HAuCl_4_ − 3 H_2_O and 33% ethanol. The surface of freshly etched silicon is covered with Si-H species, which can reduce metal ions. Thereby a nanostructured gold layer is formed on top of the porous silicon film. This galvanic displacement reaction was followed by reflectance spectroscopy using an optical fiber reflectance spectrometer (OceanOptics, Inc., USA). The deposition of a nanostructured gold layer could be noticed in the reflectance spectra by the appearance of a dip in the interference pattern resulting from the surface plasmon resonance of the nanostructured gold layer. After a reaction time of 7–7.5 min a sufficient dip at ~ 700 nm was observed in the inference pattern which could be exploited for sensing purposes. The reaction was stopped by removing the gold salt solution. Finally, the samples were washed with ethanol and dried in a stream of nitrogen.

#### Removal of Porous Silicon Film Underneath the Gold Nanostructure

In order to isolate the optical response of the nanostructured gold layer in the reflectance spectra from the interference pattern resulting from the porous silicon film, the porous silicon film underneath the gold nanostructure was removed. For this purpose samples were immersed overnight in a 1:1 (v:v) mixture of aqueous 0.01 mM NaOH solution and ethanol. Afterwards the samples were rinsed carefully with ethanol and dried slowly by leaving them exposed to air.

#### Scanning Electron Microscopy

Samples were characterized by high resolution scanning electron microscopy in order to gather information concerning the morphology of gold nanostructures, the size and shape of pores in prepared porous silicon films as well as the thickness of the layers. A scanning electron microscope from Hitachi (model S-4800) was used for taking the micrographs, which was operated at an accelerating voltage of 2.0 keV. Secondary electrons were detected by an Everhart-Thornley detector. Prior to scanning electron microscope characterization the samples were coated with carbon (5 nm) using an automatic sputter rotary coater from Quorum Technologies (model Q150R ES).

#### Interferometric Reflectance Spectroscopy

Reflectivity spectra were measured using an Ocean Optics, Inc. (USA) charged-coupled device (CCD) spectrometer (model Flame). A bifurcated optical fiber was equipped with a microscope objective lens and connected to the spectrometer as well as to a tungsten light source. Light was guided to the sample surface through the microscope objective lens illuminating a spot with a size of ~ 1–2 mm^2^. Reflectivity spectra were collected in the wavelength range of 400–1,000 nm with a spectral acquisition time of 5.7 ms. Five spectral scans were typically averaged leading to a total integration time of ~ 10 s. The illumination of the sample and the detection of the reflected light were both carried out along an axis coincident with the surface normal. In general, a reflectivity spectrum of an aluminum mirror was first collected (reference) and afterwards a reflectivity spectrum of the respective sample was recorded. The presented reflectance spectra were obtained by dividing these two spectra.

#### Determination of Refractive Index

Refractive indices of ethanol, 2-propanol, and toluene were determined at eight different wavelengths using an ATR-L refractometer from SCHMIDT + HAENSCH GmbH & Co (Germany). A Cauchy function was least square fitted to the measured values. The refractive index was then calculated using this function for the region of interest to obtain accurate values.

#### Interaction of Thiol-Terminated Polystyrene With Sensors

Sensors (porous silicon films with and without gold nanostructures on top) were fixed in a Teflon cell (prior used for etching Si wafer pieces) and reflectivity spectra were collected every 10 s using an Ocean Optics Flame CCD spectrometer throughout the experiment. After establishing a stable baseline, the Teflon cell was filled with 2.8 mL of toluene and covered with a glass slide. Finally, after 10 min 0.2 mL of a solution of thiol-terminated polystyrene in toluene was added and mixed twice. The concentration in the added 0.2 mL solution of thiol-terminated polystyrene in toluene was adjusted in order to reach a total concentration of thiol-terminated polystyrene of 1, 5, and 10 μM in the 3 mL solution covering the sensors. For determining the spectral position of the LSPR peak throughout the experiment, the LSPR peak in all acquired optical spectra was fitted to a Gaussian function.

#### Raman Spectroscopy

Raman spectra were recorded using a Witec alpha 300 confocal Raman microscope. The laser with a wavelength of 785 nm was operated at a laser power of 1 mW. The laser was focused with a Nikon 10 x plan objective (N.A. = 0.25) on the sample surface. Spectra were measured with an acquisition time of 10 s (number of accumulations: 10).

## Results and Discussion

### Fabrication of Sensors

**Figure 1:**
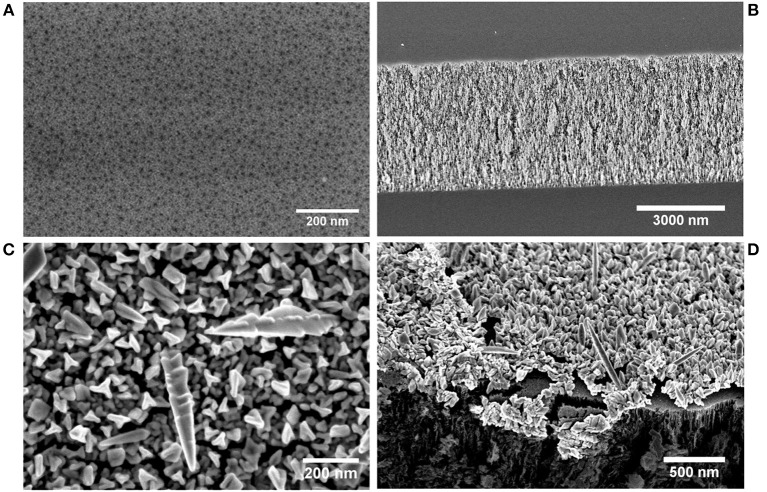
Secondary electron micrographs of the fabricated sensors. **(A)** Top view of a freshly etched PSi layer. **(B)** Cross-section of a freshly etched PSi layer. **(C)** Top view of gold nanostructures grown on a freshly etched PSi layer. **(D)** Secondary electron micrograph of gold nanostructures on a PSi layer taken with a 45° tilt angle.

Porous silicon (PSi) monolayers were fabricated by anodization of boron-doped ‹100›-oriented single-crystal Si wafers with low resistivity (0.001–0.002 Ω cm^−1^) in ethanolic HF solution. In Figure 1A a top-view scanning electron micrograph (SEM) of the resulting PSi is displayed. The prepared PSi is characterized by a sponge-like porous surface typical for p-type silicon wafers and pores with diameters below ~ 20 nm. The thickness of the PSi layer was determined by taking cross-sectional SEMs (Figure 1B) and was 4.34 μm ± 0.05 μm. The porosity of the freshly etched samples was 54.5% and was calculated by using the well-established SLIM method (Sailor, 2012). The PSi substrates were either directly used as sensors or covered beforehand with a porous gold nanoparticle layer. For the latter purpose, freshly etched PSi layers were directly immersed in a 2 mM solution of HAuCl_4_ · 3H_2_O in an ethanol/water mixture (33 % ethanol). PSi is composed of elemental silicon covered with Si-H species which enable the reduction of many metal ions by a galvanic displacement reaction (Harraz et al., 2002). The appearance of the resulting metal nanostructures depends on several reaction parameters including composition of the metal salt solution, temperature, and reaction time. Figure 1C shows a top view SEM of a PSi layer covered with gold nanostructures after immersion plating for 7 min. Deposited gold nanostructures possess different morphologies ranging from nanotripods to nanoneedles in accordance to literature (Lahiri and Kobayashi, 2016). Cross-sectional SEMs demonstrate the formation of a relatively thick layer of gold nanostructures (up to 200 nm) on top of the PSi layer (Figure 1D). Deposition of gold in the pores was not observed. In order to investigate the sensing properties of the gold nanostructure exclusively, the PSi layer underneath the gold nanostructure was dissolved by overnight immersion of the sample in an 0.01 mM NaOH solution.

### Optical Properties of Sensors

**Figure 2:**
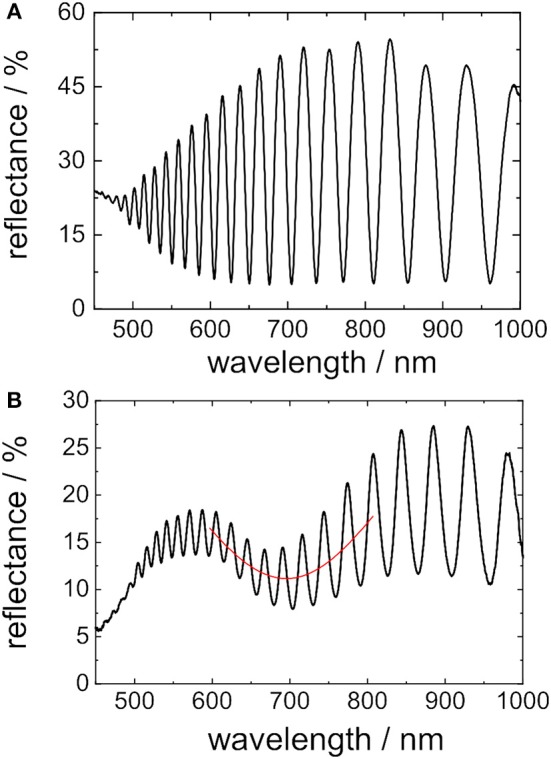
Reflectance spectra of porous silicon films without and with gold nanostructures taken at normal incidence. **(A)** Spectrum of a freshly etched porous silicon film before being immersed in gold salt solution. **(B)** Spectrum of a freshly etched porous silicon film which has been immersed in gold salt solution for 7 min - leading to the deposition of gold nanostructures on the porous silicon film.

**Figure 3:**
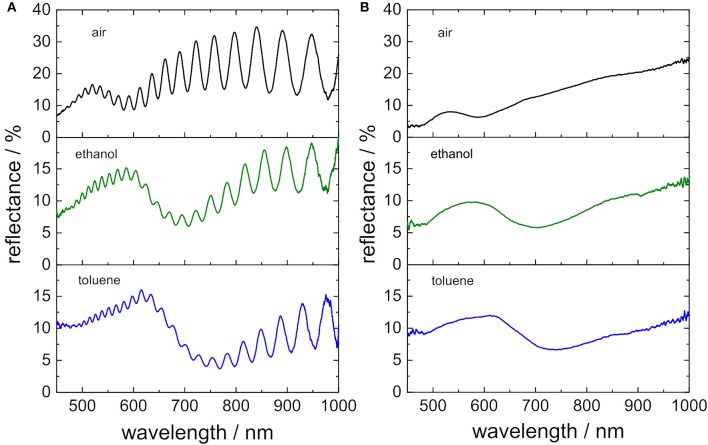
Optical characterization of sensors composed of porous silicon/gold nanostructures and gold nanostructures on silicon. **(A)** Reflectance spectra of porous silicon/gold nanostructures immersed in different dielectric media. **(B)** Corresponding reflectance spectra of isolated gold nanostructures on silicon.

In Figure 2A a representative reflectance spectrum of a porous silicon layer before deposition of gold nanostructures is shown. Typical Fabry-Pérot fringes resulting from constructive and destructive interference of light reflected at the PSi layer interfaces can be observed. After immersing the porous silicon substrate for 7 min in the gold salt solution, the reflectance spectrum is characterized by an additional broad dip in the interference pattern (Figure 2B) in the wavelength range of ~674 nm ± 22 nm (when the samples are still immersed in gold salt solution). The broad dip, referred to as Au/PSi minimum in the following text, can be attributed to the excitation of LSPR in the grown gold nanostructure. Similar observations have already been reported for silver nanoparticles on mesoporous silicon (Virga et al., 2012). The excitation wavelength (λ) of LSPR in gold nanostructures strongly depends on their size, morphology, and external dielectric environment (Stewart et al., 2008). The influence of the external dielectric media may be demonstrated by immersing the gold nanostructure in organic solvents with different refractive index (*n*) as shown in Figure 3. The Au/PSi minimum in the reflectance spectrum of the porous silicon layer covered with gold nanostructures in air (*n* = 1) is located at ~ 590 nm. After being submerged in ethanol (*n* = 1.3611) and toluene (*n* = 1.497) the Au/PSi minimum shifts to higher wavelengths, namely to ~ 708 and ~ 746 nm, respectively (Figure 3A). To isolate the optical response of the gold nanostructure from that coming from the porous silicon layer, the porous layer was dissolved by immersion of the sample in 0.01 mM NaOH solution for several hours. Thereby, the continuous Fabry-Pérot fringes typical of the reflectance spectrum of a porous silicon layer disappear and leave a signal that corresponds only to the absorption/scattering of the gold nanostructure (Figure 3B). The position of the minima found in the reflectance spectra of these isolated gold nanostructures match with the positions of the Au/PSi minima for different dielectric surrounding media.

### Sensitivity of Sensors

The bulk sensitivity of an optical sensor is defined as the shift of the optical signal λ_excitation/reference_ on the wavelength scale in nm per refractive index unit (*RIU*) (McFarland and Van Duyne, 2003; Hicks et al., 2005) and can be calculated from optical spectra of the sensor, which have been recorded in dielectric media with different refractive indices. Here, the position of the optical signal of the sensor, e.g., a specific interference maximum or LSPR, shifts to longer wavelengths (λ) as the refractive index (*n*) of the medium increases. Therefore, the sensitivity of an optical sensor may be determined by plotting the position of the signal on the wavelength scale in different media or the magnitude of the shift (λ_*n*≠1_ − _*n* = 1_) vs. the *n* of the dielectric medium. The slope of the resulting calibration curve is the magnitude of bulk sensitivity of the sensor. The developed optical sensor, composed of a PSi layer covered with gold nanostructures, has two different transduction signals, namely interference and LSPR, whose bulk sensitivities were investigate in depth and will be presented in the following.

#### Porous Silicon: Interferometry

**Figure 4:**
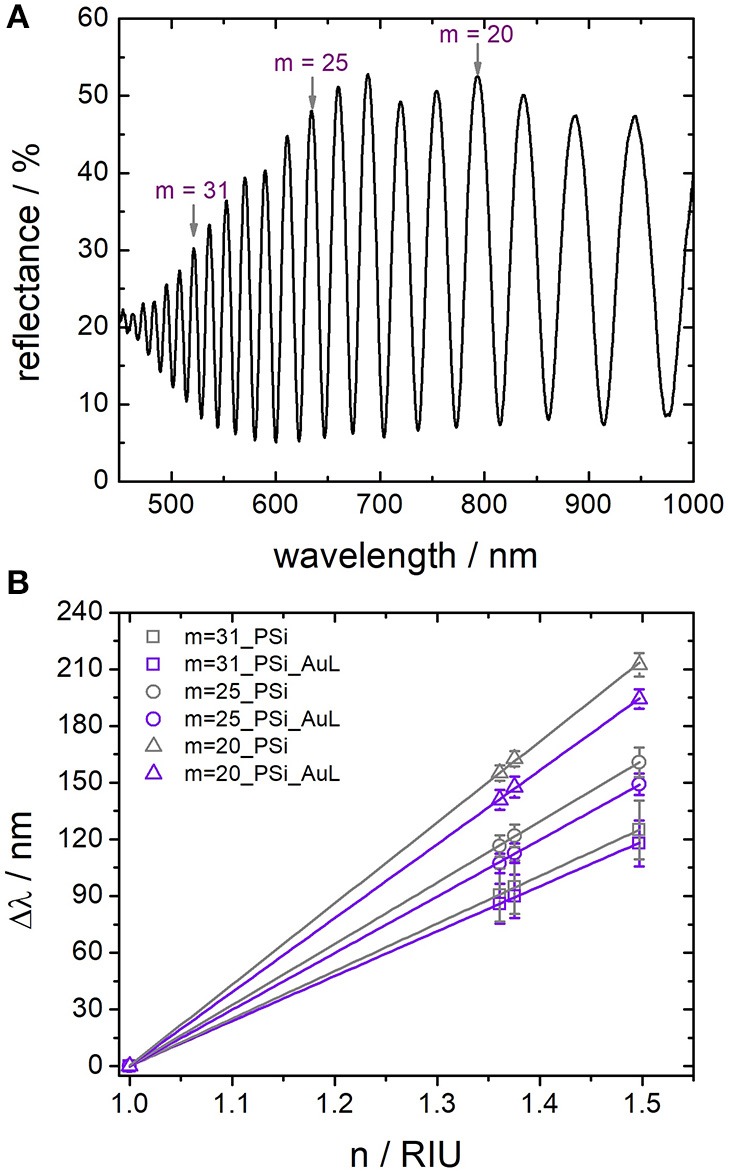
**(A)** Reflectance spectra of a porous silicon sensor in air. The spectral order (*m*) refer to the interference maxima which were investigated in order to calculate sensitivities. **(B)** Determination of the sensitivity of sensors by plotting the position of single interference maxima on the wavelength scale vs. the refractive index of the surrounding medium.

In Figure 4A a representative reflectance spectrum of a PSi layer taken at normal incidence showing the typical interference pattern is displayed. The interference maxima are located at wavelengths λ_*m*_ which fulfill the Fabry-Pérot relationship:

(1) $$\begin{array}{l} \lambda_{m}=\;\frac{2n_{ef}L}{m} \end{array}$$

where *m* is the spectral order of a fringe, *L* is the physical thickness in nm, and *n*_*ef*_ is the refractive index of the effective medium of the porous layer. Hence, the position of an interference maximum λ_*m*_ is proportional to the refractive index of the surrounding medium *n*_*ef*_. In order to evaluate the influence of the gold nanostructure on the optical response of the PSi layer, two different sensors, namely a freshly etched PSi sensor with and without gold nanostructures on top, were immersed in different organic solvents and reflectance spectra were recorded. In this study ethanol, 2-propanol, and toluene were used whose refractive index was determined at the wavelength matching the position of the interference maximum under investigation. First, shifts of three interference maxima, which are located approximately at 520 nm, 640 nm and 790 nm at *n* = 1, were considered. The term *2n*_*ef*_*L* in Equation (1) is the so-called effective optical thickness (EOT) and can be calculated by plotting *m* vs. 1*/*λ_m_ (*m* = is the spectral order of a fringe and λ_m_ = the wavelength at which each interference maximum appears) resulting in a straight line whose slope is equal to the EOT. Another convenient way to calculate the EOT is the application of a fast Fourier transform (FFT) to the reflectance spectrum in wavenumbers, even though this approach neglects the frequency dispersion of the refractive index as well as multiple reflections (Sailor, 2012). The application of the FFT to the interference spectra results in a graph of “intensity vs. EOT” which displays a peak at the position of the highest intensity corresponding to the magnitude of the EOT in nm. By using this technique to calculate the EOT it is possible to estimate *m* at *n* = 1:

(2) $$\begin{array}{l} m=\;\frac{EOT}{\lambda_{m}} \end{array}$$

Therefore, to the interference maxima located at 520, 640, and 790 nm correspond spectral orders of *m* = 31, *m* = 25 and *m* = 20, respectively Similarly, it is possible to calculate the EOT of the structures after immersion in the organic solvents to estimate the λ_*m*_ that corresponds to each *m*. By choosing these interference maxima at *n* = 1 the effect of the LSPR dip on the interference pattern at varying wavelengths should be evaluated. In Figure 4B a plot of the wavelength shifts (Δλ = λ_m*atn*≠1_ − λ_m*atn* = 1_) of the three interference maxima vs. the refractive index of the solvents at these wavelengths is displayed. The responses of PSi sensors with and without gold nanostructures on top are shown in violet and gray, respectively. A strong influence of a gold nanostructure deposited on a PSi sensor on the sensitivity of the porous silicon was not observed for the investigated interference maxima at different wavelengths. The sensitivity of the interference maxima with spectral orders of *m* = 31, *m* = 25, and *m* = 20 in the spectrum of PSi without the nanostructured gold layer was (252 ± 1), (324 ± 1), and (429 ± 3) nmRIU^−1^, respectively. Similar sensors composed of PSi with gold nanostructures on top provided sensors with sensitivities of (238 ± 1), (300 ± 2), and (391 ± 1) nm RIU^−1^, which are slightly lower than the values for unmodified porous silicon—as expected due to the refractive index dispersion.

**Figure 5:**
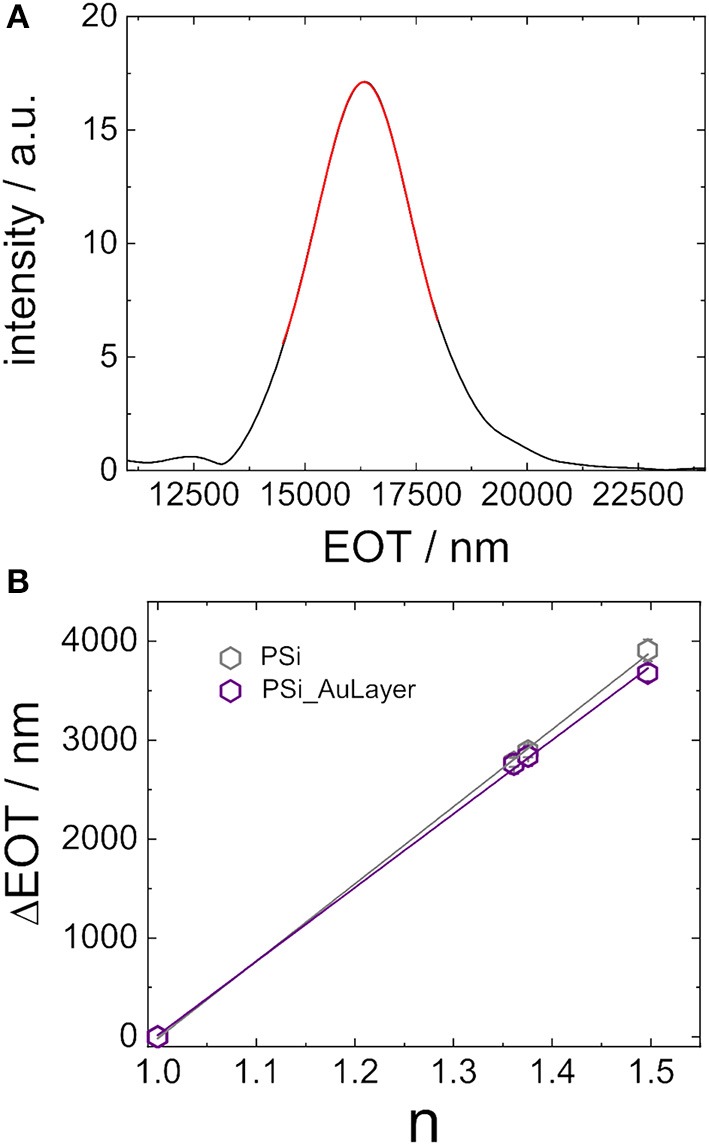
**(A)** Effective optical thickness of sensors in air calculated by applying a fast Fourier transform to appropriately prepared reflectance spectra. **(B)** Sensitivity of sensors determined by using shifts in the EOT in response to changing the refractive index of the immersion medium.

The examination of shifts of interference maxima on the wavelength scale only provides a rough estimation of the optical sensor responses. Most often the EOT, is exploited for interferometric sensing with PSi sensors. In Figure 5A a representative fast Fourier transform of a PSi layer is displayed. The position of the peak in the Fourier transform is then determined by fitting a Gaussian function to it, indicated by the red line, and represents the EOT of the porous silicon layer. The EOT peak shifts to smaller or to larger values when the refractive index of the porous matrix is modified, i.e., upon filling of the pores with different media, surface modification, or molecular adsorption. Again, the sensitivity of both PSi sensors with (in violet) and PSi sensors without gold nanostructure (in gray) on top are examined. Figure 5B shows the corresponding plot of ΔEOT = EOT_n≠1_ –EOT_n = 1_ vs. the refractive index of the used organic solvent. Again, a linear relationship between EOT and refractive index is obtained whose slope was determined to be 7,812 nm RIU^−1^ ± 113 nm RIU^−1^ and 7,463 nm RIU^−1^ ± 135 nm RIU^−1^ for the porous silicon sensors with and without gold nanostructures on top, respectively. These values are in accordance with porous silicon sensors Specifically, for interferometric sensors using EOT as transduction signal, it is important to highlight that the sensitivity (S) may be defined as:

(3) $$\begin{array}{l} S=\;\frac{dEOT}{dn}=2L\frac{dn_{ef}}{dn} \end{array}$$

Hence, the sensitivity of an interferometric sensor might be amplified by the value of the thickness of the layer, however, for PSi, the diffusion of analytes inside the pores also plays an important role.

#### Gold Nanostructure: LSPR

**Figure 6:**
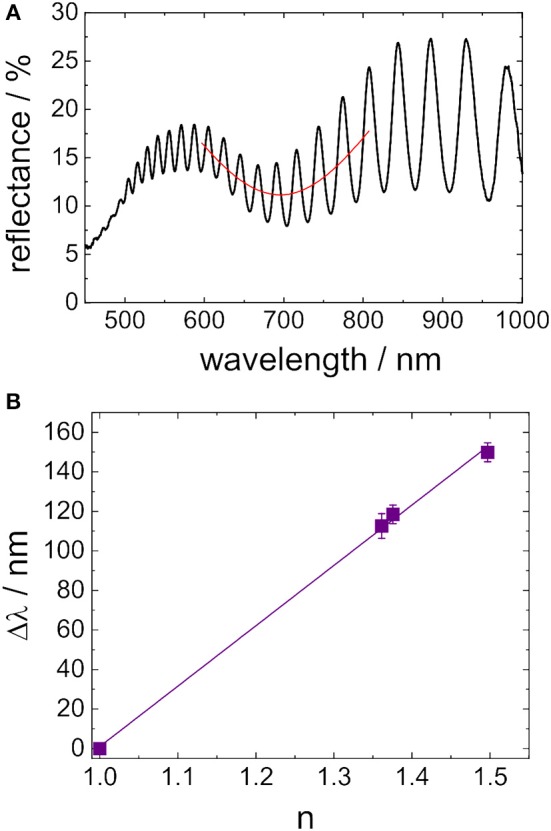
**(A)** Reflectance spectra of hybrid sensors composed of gold nanostructures and porous silicon (red lines are integrated in order to guide the eye to the LSPR feature in the spectrum). **(B)** Plot of the position of LSPR on the wavelength scale vs. the refractive index of the medium surrounding the sensor.

In Figure 6A a representative reflectance spectrum of a PSi sensor with gold nanostructures on top is shown. The broad dip in the interference pattern is assigned to LSPR of the gold nanostructure and its position was estimated by a least square fit to an Gaussian function (invers), indicated as a red line in the spectrum. In general, the position of LSPR on the wavelength scale λ_*LSPR*_ strongly depends on the refractive index *n* of the surrounding medium. Figure 6B shows a plot of Δλ_LSPR_ (Δλ_LSPR_ = λ_LSPR, n≠1_ − λ_LSPR, n = 1_) vs. the refractive index *n* of the surrounding medium. The expected linear relationship was obtained. The bulk sensitivity of the gold nanostructure was 307 nm RIU^−1^ ± 8 nm RIU^−1^. This value is in the upper region for LSPR sensing (Mayer and Hafner, 2011).

Nevertheless, the magnitude of the sensitivity does not define by itself the capacity of the sensor to detect and quantify an analyte. An ideal refractive index-based sensor should be capable to quantify the smallest change in *n* due to the emerging or increase in concentration of an analyte. However, a lot of parameters have an influence on the sensor performance including the properties of the instrumentation used to measure (spectrometer, temperature control, etc.) and the method used to process the experimental data. For example, the concept of resolution (*R*) was presented to define the smallest variation of the surrounding refractive index that a sensing device is able to resolve (Chiavaioli et al., 2017). It is important to emphasize that there is still a discordance about the way how to evaluate the magnitude and the units of *R*. In literature values of *R* are expressed in longitude units (nm or pm) or RIU, depending on the chosen method for calculating this value. *R* is given in longitude units when it is calculated based on the standard deviation (σ) of the noise in the system (White and Fan, 2008). A more recent approach has defined R as:

(4) $$\begin{array}{l} R=\frac{p\sigma }{S} \end{array}$$

where *p* represents the confidence interval. A relatively rough approach was chosen in this study for estimating *R*. It was defined as the standard deviation of the experimental points acquired for obtaining a baseline in the sensing experiments, carried out in air and in toluene, divided by *S*. The average standard deviation of the experimental baselines were 0.06 nm ± 0.01 nm and 0.36 nm ± 0.14 nm in air and toluene, respectively. Therefore, *R* of the nanostructured gold layer has magnitudes of 2.03x10^−4^ RIU and 1.16x10^−3^ RIU in air and in toluene, respectively. Another inconsistency in the evaluation and comparison of different sensors resides in the similarity of Equation (5) with a known equation describing the detection limit (DL):

(5) $$\begin{array}{l} DL=\frac{R}{S} \end{array}$$

The use of Equation (6) usually describes R as the standard deviation of the spectral measurement as explained above. In this case, R and DL can be considered as synonyms (Amore et al., 1980; Mariani et al., 2018b). However, it has been claimed that Equation (6) does not accurately calculate the DL (Loock and Wentzell, 2017). The DL should indicate the smallest amount of analyte that causes a refractive index change that can be accurately quantified by a device. It should be expressed in terms of concentration. As different analytes might have a different influence on the same sensing surface, the DL must be calculated for each particular case in a label free sensor. In the presented study the DL of thiol-terminated polystyrene will be determined.

Due to the fact that optimum sensitivity and resolution of plasmonic sensors is sometimes related to the width of the optical signal and not to the magnitude of the refractive index shift, Sherry et al. (2005) introduced the concept of figure of merit (FOM). It allows for a more accurate comparison between the sensing capacity of different plasmonic nanostructures, and is defined as:

(6) $$\begin{array}{l} FOM=\frac{S}{fwhm} \end{array}$$

where *fwhm* represents the full width at half-maxima of the plasmon peak in the spectrum. The LSPR peak in the spectrum had a *fwhm* value of 79 nm ± 11 nm, hence, the FOM of the plasmonic sensor is 3.88 in air.

**Figure 7:**
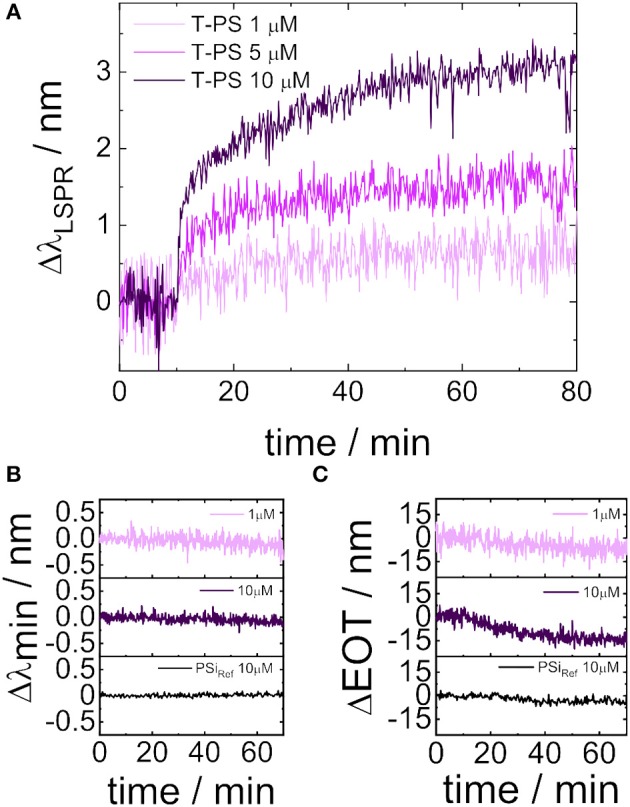
Real-time monitoring of the interaction of thiol-terminated polystyrene with the hybrid sensor comprising two different sensor surfaces (gold nanostructure / porous silicon). **(A)** Shift in the LSPR wavelength of the gold nanostructure in response to immersing the sensor in solutions of thiol-terminated polystyrene in toluene (three different concentrations). **(B)** Changes in the position of an interference minimum located at ~ 741 nm in toluene upon addition of thiol-terminated polystyrene. **(C)** Changes in the effective optical thickness of a reference porous silicon film in response to thiol-terminated polystyrene in the immersion medium.

However, LSPR is only sensitive to refractive index changes close to the dielectric/metal interface and the following relationship is the basis for LSPR wavelength-shift sensing:

(7) $$\begin{array}{l} {\Delta \lambda }_{LSPR}=m\Delta n\left[1-exp\left(\frac{-2d}{l_{d}}\right)\right] \end{array}$$

where *m* is bulk refractive index response of the gold nanoparticles, Δ*n* is the refractive index changed caused by the adsorbate, *d* is the effective adsorbate layer thickness on the nanoparticle surface, and *l*_*d*_ is the EM-field-decay length (Willets and Van Duyne, 2007). The high sensitivity for detecting analytes adsorbed at the sensor surface is a result of the short decay length of the plasmonic field in the case of LSPR (usually below ten nanometers), i.e., a large fraction of the analyte is concentrated in the total sensing volume. As a result LSPR sensors are perfectly suitable for monitoring adsorption of molecules on the gold nanostructure in real-time. Also, the presented sensor, composed of PSi and a gold nanostructure on top, is capable of detecting adsorption processes. To demonstrate this capability, the sensor was first immersed in toluene in order to establish a baseline and subsequently a small amount of a solution of thiol-terminated polystyrene in toluene was added. The thiol group at the end of the polystyrene chain chemisorbs at the gold nanostructure. The surface coverage of the gold surface with the thiol-terminated polystyrene depends on several parameters including the length of the polystyrene chains and the concentration of the thiol-terminated polystyrene in solution (Stouffer and McCarthy, 1988). In this study, thiol-terminated polystyrene possessing a molecular weight of 31,400 g mol^−1^ was used in order to achieve both a good surface coverage of the gold and an exclusion of the polystyrene from the PSi layer. The adsorption process was monitored by recording reflectance spectra at normal incidence every 10 s. The shift of the LSPR peak position on the wavelength scale due to binding of thiol-terminated polystyrene to the gold surface over time is displayed in Figure 7A. To compare the optical responses of different sensors the shift was calculated using the following equation:

(8) $$\begin{array}{l} {\Delta \lambda }_{LSPR}={LSPR}_{t\neq 0}-{LSPR}_{t=0} \end{array}$$

Where *LSPR*_*t*=0_ and *LSPR*_*t*≠0_ are the LSPR peak position before and after addition of thiol-terminated polystyrene, respectively. The adsorption process was investigated for thiol-terminated polystyrene solutions with varying concentrations. At a low concentration of thiol-terminated polystyrene in toluene (1μM) a small shift of < 1 nm was measured and steady state was reached in < 60 min. Δ*LSPR* increased proportional when the initial concentration of the thiol-terminated polystyrene solution was raised (5 μM and 10 μM). These results are in accordance with reported investigations on the adsorption kinetics of thiol-terminated polystyrene on gold surfaces and could be described by Langmuir isotherms (Stouffer and McCarthy, 1988).

In order to demonstrate that the adsorption of thiol-terminated polystyrene to the gold nanostructure is exclusively monitored by shifts of the LSPR peak to longer wavelengths and not by changes in the interference pattern of the PSi layer underneath, the reflectance spectra of the sensor are thoroughly analyzed. In Figure 7B changes in the position of the interference minimum located at ~ 741 nm, in the middle of the broad dip resulting from LSPR, during the adsorption process are shown. A shift of the interference minimum to longer wavelengths was observed neither at high nor at low thiol-terminated polystyrene concentrations indicating the exclusion of the thiol-terminated polystyrene from the pores. This result was confirmed by reference measurements using a PSi layer without gold nanostructures on top (PSi_Ref_). Also, in Figure 7C an increase in the EOT of the PSi structure underneath the gold layer could not be detected in response to the addition of thiol-terminated polystyrene to toluene surrounding the sensor.

Finally, in order to calculate the DL of the system (using thiol terminated polystyrene as analyte) a method based on a calibration curve of the sensor was used - as has been explained by Chiavaioli et al. (2017). This method considers three times the standard deviation of blank measurements (3σ, blank measurement is referred to measurements without the analyte under investigation), the mean value of the blank measurement (*Y*_blank_), a calibration curve of the sensor evaluated at a concentration of roughly 1–5 times higher than the suspected DL and an accurate fitting function of such calibration curve (linear, parabolic, exponential, etc.). Then, the following equation can be applied:

(9) $$\begin{array}{l} x_{DL}=f^{-1}\left({\text{Y}}_{blank}+3\sigma \right) \end{array}$$

where *x*_DL_ (concentration of the detection limit) is equal to the inverse of the function (*f* ^−1^) evaluated at (*Y*_blank_ +3σ). The presented senor is reaching its DL at a concentration of 1 μM of thiol terminated polystyrene, as can be deduced from Figure 7A. The theoretical DL was calculated using the data, which were obtained from Equation (9). The *Y*_blank_ has a normalized value of 0. The calibration curve of the sensor can be fitted to a parabolic equation. The DL concentration for thiol terminated polystyrene using the gold nanostructure on top of a porous silicon layer was determined to be 0.328 μM.

### Surface Enhanced Raman Spectroscopy

**Figure 8:**
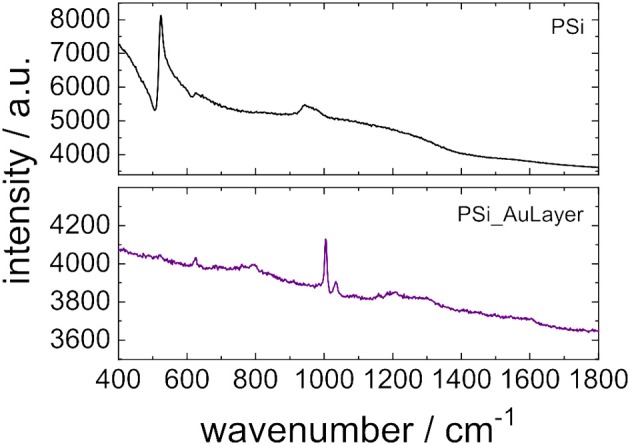
Raman spectra of thiol-terminated polystyrene deposited on porous silicon films without **(A)** and with gold nanostructure on top **(B)**.

Sensors composed of PSi layers with gold nanostructure on top were tested as substrates for SERS. For this purpose, the sensors were immersed in a 10 μM solution of thiol-terminated polystyrene (*M* = 31,400 g mol^−1^) in toluene for 3 h and subsequently dried at room temperature. A PSi layer without gold nanostructure served as reference and was treated in the same way. In Figure 8 Raman spectra of PSi based sensors without and with gold nanostructure after functionalization with thiol-terminated polystyrene are displayed. The Raman spectrum of the PSi layer without gold shows a band at 523 cm^−1^ and another broad band ranging from 930–990 cm^−1^ which both are both characteristic for porous silicon (Figure 8A) (Tanino et al., 1996). However, no Raman bands of polystyrene can be identified. Their absence in the Raman spectrum can be interpreted in two different ways. On the one hand, only a small amount of thiol-terminated polystyrene may have been deposited on the porous silicon surface, which supports the assumption of exclusion of thiol-terminated polystyrene from the pores. On the other hand Raman scattering is in general a rather weak effect and the detection limits for molecules by Raman spectroscopy are relatively high. In the case of the PSi layer with gold nanostructures on top the Raman scattering is greatly enhanced and the Raman spectrum clearly shows a strong band at 1,003 cm^−1^ which is characteristic for expansion and contraction of the phenyl rings in polystyrene (Figure 8B) (Mccaffery and Durant, 2003). Furthermore, bands at 623 and 1,034 cm^−1^ can be noticed in the Raman spectrum which can be assigned to the ν_6b_ and ν_18a_ vibrations of polystyrene, respectively (Pollard et al., 2017). The standard deviation of the SERS measurements was 14 % and was determined by analyzing Raman spectra which were recorded at five different spot distributed over a sensor surface of 1.13 cm^2^.

## Summary

In a nutshell, PSi monolayers were electrochemically prepared and subsequently decorated with gold nanostructures by immersion of freshly etched PSi in a solution containing gold ions. The deposition reaction was monitored by reflectance spectroscopy and the appearance of a broad dip at ~600 nm was observed. This dip can be assigned to LSPR of the gold nanostructures on top of the porous layer – nanostructured gold layer particles deposited directly on Si showed a broad peak at approximately the same wavelength. Thorough investigations on the sensing performance of these structures were conducted using reflectance spectroscopy. The bulk sensitivity of the gold nanostructure was determined to be higher in comparison to the one of PSi if changes in the positions of single interference minima on the wavelength scale were considered. Moreover, the adsorption of thiol-terminated polystyrene to the sensor surface could only be monitored by shifts in the LSPR peak to longer wavelengths suggesting independence of interferometric and LSPR sensing with the presented optical sensor.

## Data Availability

All datasets generated for this study are included in the manuscript.

## Author Contributions

RB-V fabricated and characterized all nanostructures, as well as carried out the reflectivity measurements and sensor experiments. RS recorded all Raman spectra and analyzed them. CP has conceived the research and designed the study. RB-V and CP analyzed all data. All authors discussed the results and developments at all stages and wrote the manuscript.

### Conflict of Interest Statement

The authors declare that the research was conducted in the absence of any commercial or financial relationships that could be construed as a potential conflict of interest.
